# Editorial

**DOI:** 10.1093/bib/bbae453

**Published:** 2024-09-17

**Authors:** Shuangge Ma

**Affiliations:** Department of Biostatistics, Yale School of Public Health 300 George ST, Suite 501 New Haven, CT 06511, USA

It has been slightly more than a year since I took over the position of Editor-in-Chief of Briefings in Bioinformatics (BIB). When I look back, the first thing that comes to mind is great appreciation. I would like to take this opportunity to thank all readers, authors, reviewers, and the editorial team—the journal could not have stayed this strong without your fully dedicated support. BIB remains one of the most highly ranked and competitive journals in bioinformatics and a top choice for numerous bioinformatics researchers worldwide.

Similar to many other bioinformatics journals, we have witnessed a surge in submissions that involve AI, deep learning, and machine learning. BIB has become a leading venue for publishing new methods, applications, and reviews in AI and deep learning. There is no doubt that such techniques have and will continue to revolutionize science including bioinformatics. Along the way, there can be a few aspects worth some additional consideration.

Many bioinformatics datasets are perceived as being challenged by small sample sizes and high-dimensional input. It is not fully clear whether/how deep neural networks can be immune from the curse of dimensionality, which may lead to a lack of stability, inferior prediction performance, etc. Some recent techniques have grown increasingly complicated, with more complex architectures and more layers/nodes. There is a strong and increasing demand for stability evaluation metrics and techniques as well as methods that can purposely improve stability. A naturally related question is: with a certain sample size (amount of information), what is the maximum complexity a deep neural network model can have? In classic analytics, there are some rules of thumb (for example, in regression analysis 101, it is recommended that the ratio of sample size/number of input variables is at least 5–10). However, such guidelines are missing in AI and deep/machine learning-based bioinformatics studies. It is recommended that more attention to complexity, stability, limitations in training data, etc. is paid in future studies.

Different from some scientific domains, interpretability can be highly desirable in many bioinformatics studies. In classic bioinformatics, interpretability is enhanced by the relatively lucid model structures, differentiation/identification of signals from noises, quantification of effect sizes, and elucidation of causal paths. It is recognized that, with the foundational differences of AI and deep/machine learning, we need to rethink some of those perspectives. However, interpretability overall is still and may be more desirable—this view is shared by numerous research organizations, funding agencies, and researchers. We welcome more discussions/research on interpretability and the development of (more) interpretable AI/deep learning techniques.

For most if not all bioinformatics problems and datasets, there are multiple available approaches. In the process of developing new approaches, it is critical to know how they compare against existing alternatives. In practical applications, it is necessary to know when an approach works (and, equally importantly, when it does not). Rigorous theoretical investigations remain somewhat rare in bioinformatics technology development. Well-designed, extensive, and fair simulations and comparisons with state-of-the-art existing techniques can serve this purpose to a certain extent. Recognizing the limitations of synthetic data, and the ultimate goal of analyzing practical data, we recommend carefully gauged comparisons based on extensive, unbiased, and high-quality data. Studies with selection bias in data and benchmarks will not be sufficiently appealing.

“Plurality should not be posited without necessary”—Occam’s razor. Bioinformatics problems are growing more complicated and demand the development of more complex tools. On the other hand, it is also recognized that some practical problems can be equally resolved with existing tools that may be simpler, more robust, and lucid. As such, when a new (and likely more complicated) approach is developed, it is strongly recommended that it is benchmarked against existing alternatives comprehensively in terms of model fitting, computational cost, stability, and other aspects, which will better inform practitioners how to choose tools properly.

To date, BIB does not uniformly require making software and data fully publicly available. Rather, this is handled on a case-by-case basis. The public availability of software and data ensures reproducibility and facilitates broad utilization. It will be a huge burden to society if users have to re-develop software for a published technique. For data, we encourage sharing when it is feasible (for example, allowed by funding agencies), though require access to data if needed for peer review. For software, especially when it is a new and nontrivial approach, we very strongly encourage depositing at stable and publicly accessible repositories. Such recommendations have been strongly advocated by many reviewers and readers. We welcome input from all readers and authors regarding reproducibility and publication of data and software.

The field of bioinformatics has never been as dynamic as it is right now. With your support, I am fully confident of the even brighter future of BIB. Again, thank you.

